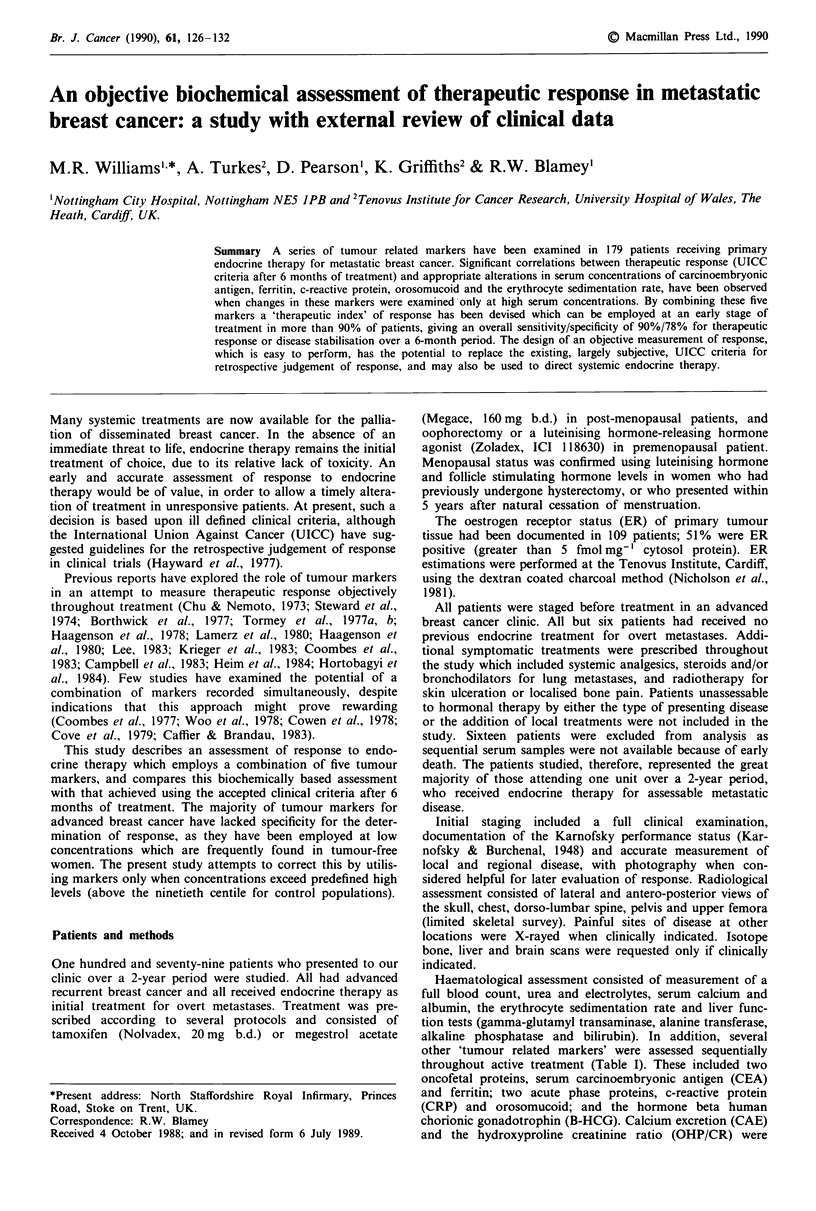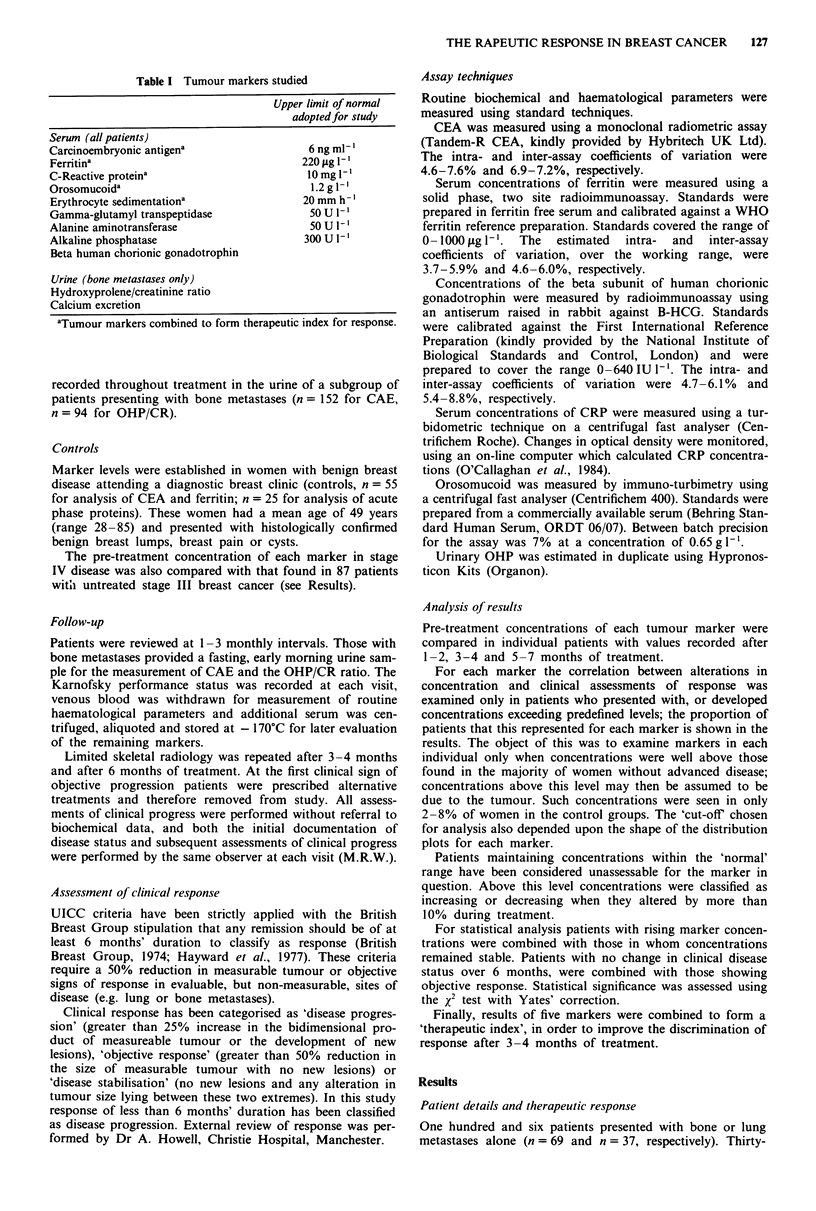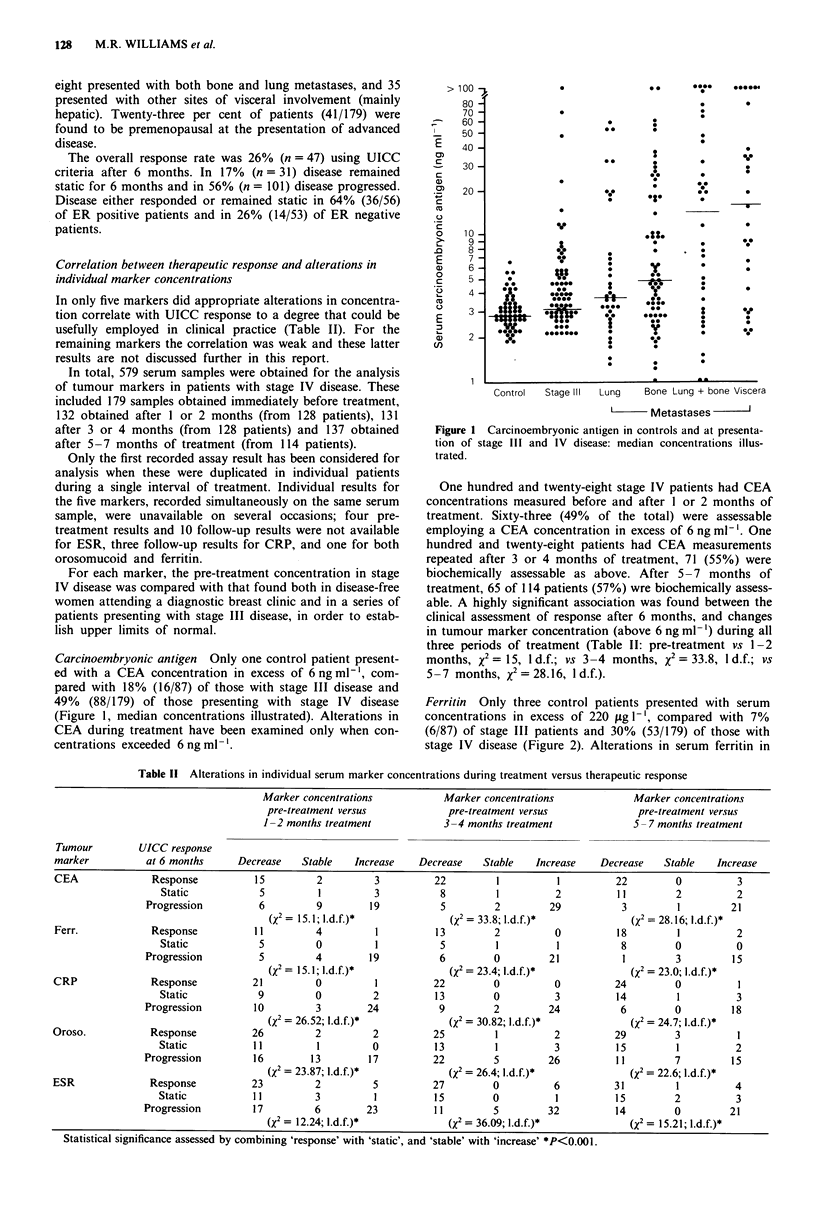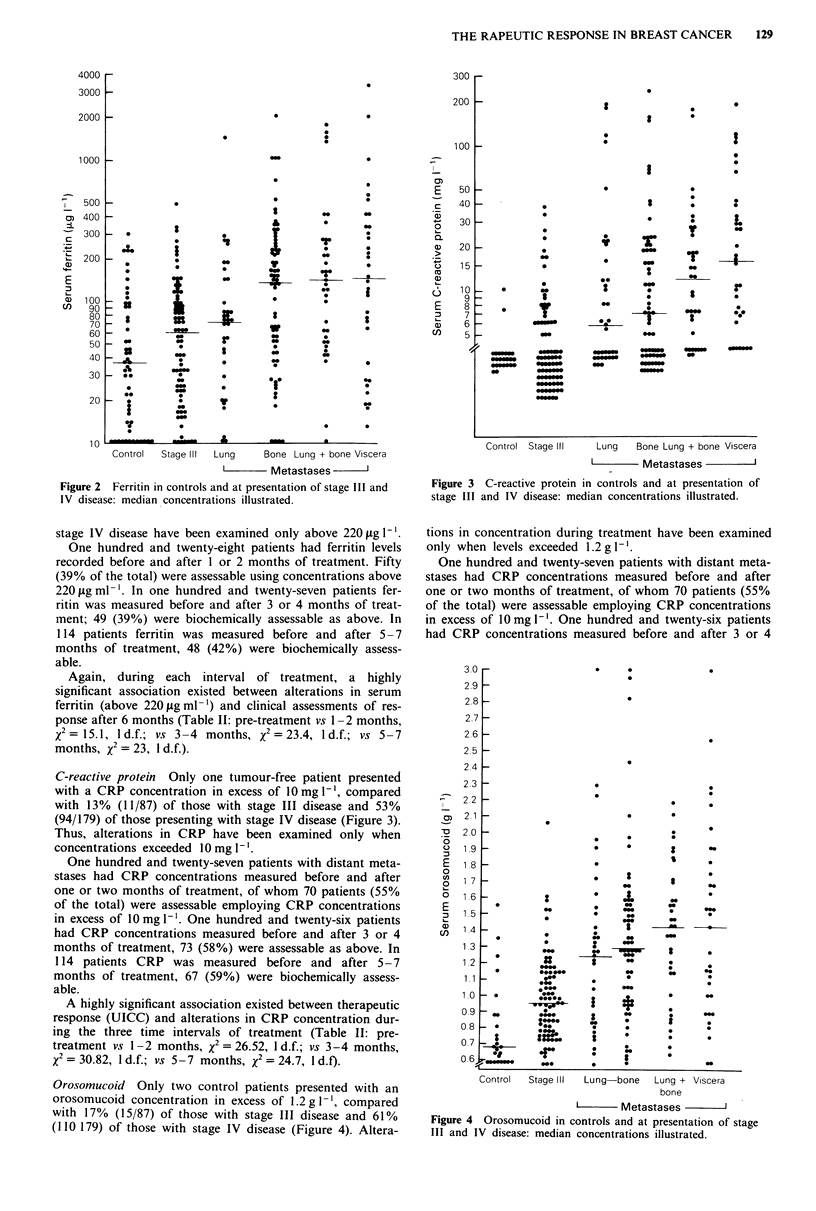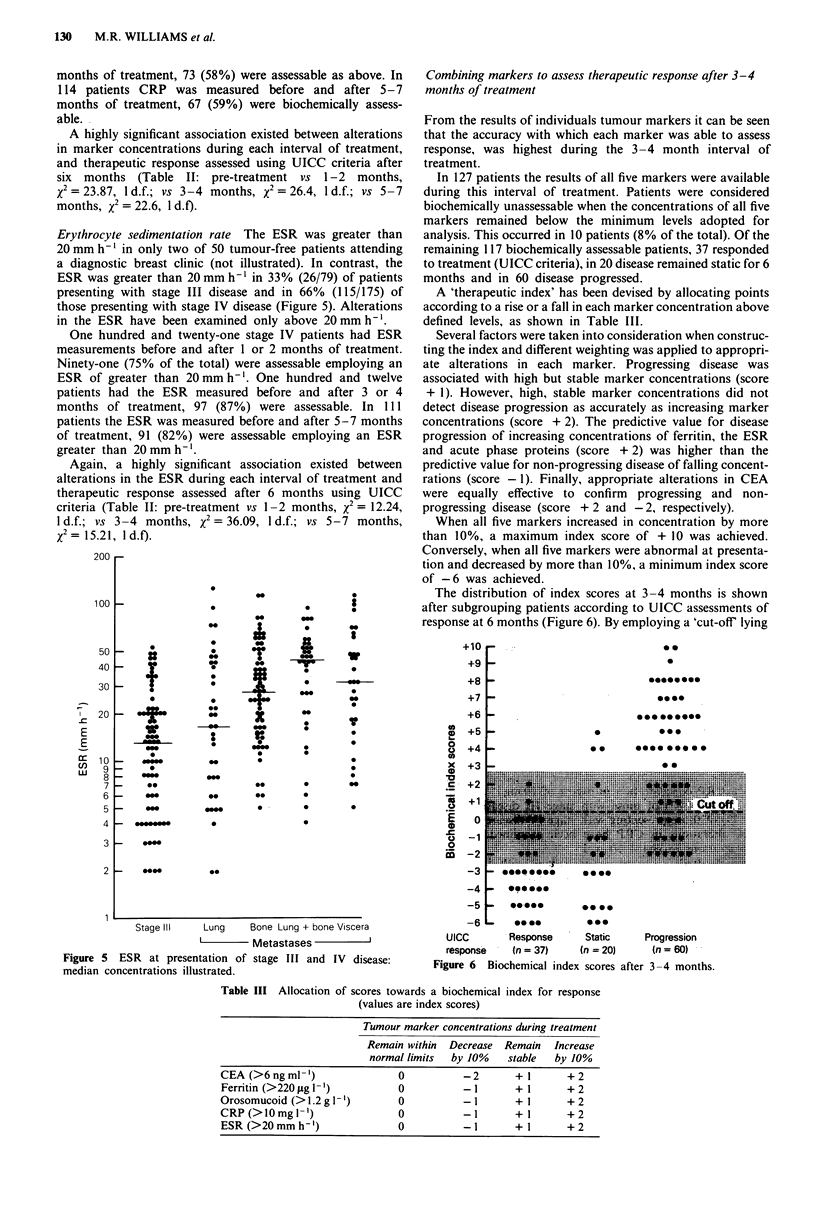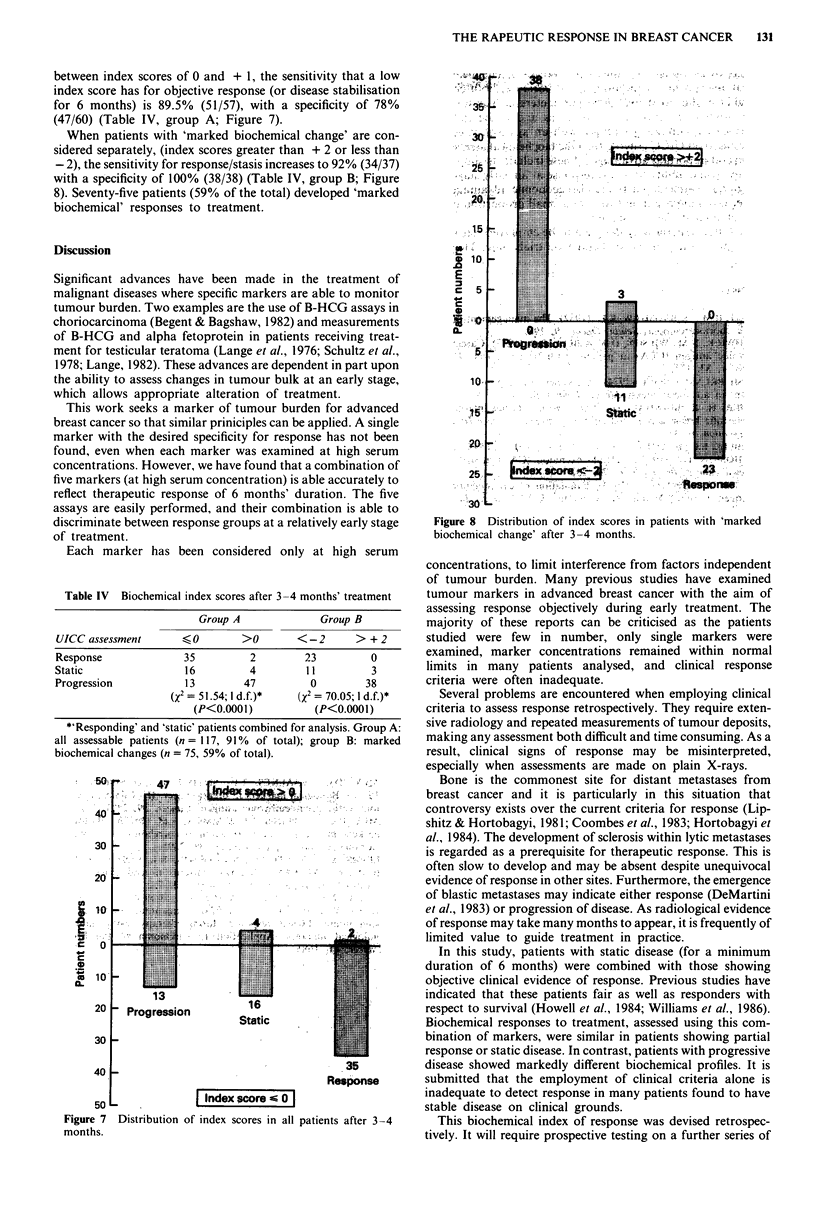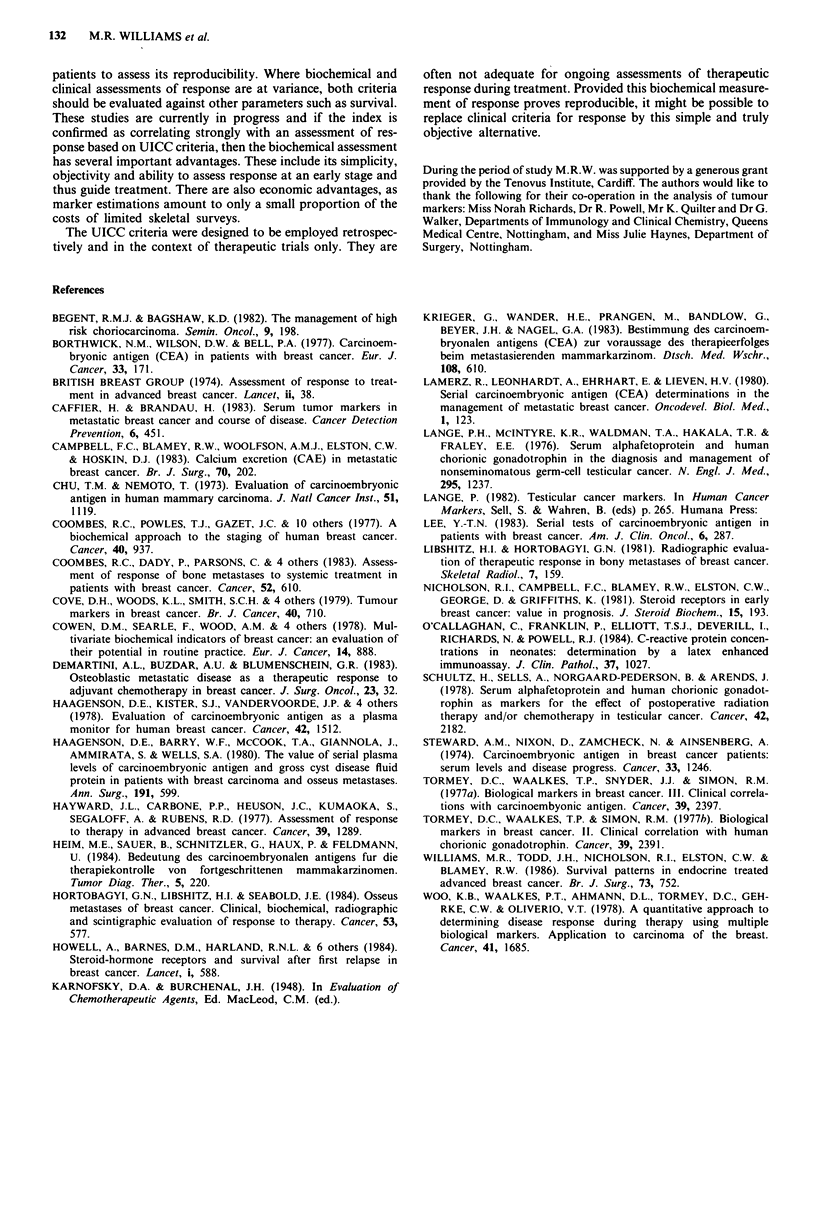# An objective biochemical assessment of therapeutic response in metastatic breast cancer: a study with external review of clinical data.

**DOI:** 10.1038/bjc.1990.26

**Published:** 1990-01

**Authors:** M. R. Williams, A. Turkes, D. Pearson, K. Griffiths, R. W. Blamey

**Affiliations:** Nottingham City Hospital, UK.

## Abstract

A series of tumour related markers have been examined in 179 patients receiving primary endocrine therapy for metastatic breast cancer. Significant correlations between therapeutic response (UICC criteria after 6 months of treatment) and appropriate alterations in serum concentrations of carcinoembryonic antigen, ferritin, c-reactive protein, orosomucoid and the erythrocyte sedimentation rate, have been observed when changes in these markers were examined only at high serum concentrations. By combining these five markers a 'therapeutic index' of response has been devised which can be employed at an early stage of treatment in more than 90% of patients, giving an overall sensitivity/specificity of 90%/78% for therapeutic response or disease stabilisation over a 6-month period. The design of an objective measurement of response, which is easy to perform, has the potential to replace the existing, largely subjective. UICC criteria for retrospective judgement of response, and may also be used to direct systemic endocrine therapy.


Br. J. Cancer (1990), 61, 126-132 © Macmillan Press Ltd., 1990

### An objective biochemical assessment of therapeutic response in metastatic breast cancer: a study with external review of clinical data

M.R. Williams[1,*], A. Turkes[2], D. Pearson[1], K. Griffiths[2] & R.W. Blamey[1]

[1]*Nottingham City Hospital, Nottingham NE5 1PB and* [2]*Tenovus Institute for Cancer Research, University Hospital of Wales, The Heath, Cardiff, UK.*

**Summary** A series of tumour related markers have been examined in 179 patients receiving primary endocrine therapy for metastatic breast cancer. Significant correlations between therapeutic response (UICC criteria after 6 months of treatment) and appropriate alterations in serum concentrations of carcinoembryonic antigen, ferritin, c-reactive protein, orosomucoid and the erythrocyte sedimentation rate, have been observed when changes in these markers were examined only at high serum concentrations. By combining these five markers a 'therapeutic index' of response has been devised which can be employed at an early stage of treatment in more than 90% of patients, giving an overall sensitivity/specificity of 90%/78% for therapeutic response or disease stabilisation over a 6-month period. The design of an objective measurement of response, which is easy to perform, has the potential to replace the existing, largely subjective, UICC criteria for retrospective judgement of response, and may also be used to direct systemic endocrine therapy.

Many systemic treatments are now available for the palliation of disseminated breast cancer. In the absence of an immediate threat to life, endocrine therapy remains the initial treatment of choice, due to its relative lack of toxicity. An early and accurate assessment of response to endocrine therapy would be of value, in order to allow a timely alteration of treatment in unresponsive patients. At present, such a decision is based upon ill defined clinical criteria, although the International Union Against Cancer (UICC) have suggested guidelines for the retrospective judgement of response in clinical trials (Hayward *et al.*, 1977).

Previous reports have explored the role of tumour markers in an attempt to measure therapeutic response objectively throughout treatment (Chu & Nemoto, 1973; Steward *et al.*, 1974; Borthwick *et al.*, 1977; Tormey *et al.*, 1977*a*, *b*; Haagenson *et al.*, 1978; Lamerz *et al.*, 1980; Haagenson *et al.*, 1980; Lee, 1983; Krieger *et al.*, 1983; Coombes *et al.*, 1983; Campbell *et al.*, 1983; Heim *et al.*, 1984; Hortobagyi *et al.*, 1984). Few studies have examined the potential of a combination of markers recorded simultaneously, despite indications that this approach might prove rewarding (Coombes *et al.*, 1977; Woo *et al.*, 1978; Cowen *et al.*, 1978; Cove *et al.*, 1979; Caffier & Brandau, 1983).

This study describes an assessment of response to endocrine therapy which employs a combination of five tumour markers, and compares this biochemically based assessment with that achieved using the accepted clinical criteria after 6 months of treatment. The majority of tumour markers for advanced breast cancer have lacked specificity for the determination of response, as they have been employed at low concentrations which are frequently found in tumour-free women. The present study attempts to correct this by utilising markers only when concentrations exceed predefined high levels (above the ninetieth centile for control populations).

### Patients and methods

One hundred and seventy-nine patients who presented to our clinic over a 2-year period were studied. All had advanced recurrent breast cancer and all received endocrine therapy as initial treatment for overt metastases. Treatment was prescribed according to several protocols and consisted of tamoxifen (Nolvadex, 20 mg b.d.) or megestrol acetate (Megace, 160 mg b.d.) in post-menopausal patients, and oophorectomy or a luteinising hormone-releasing hormone agonist (Zoladex, ICI 118630) in premenopausal patient. Menopausal status was confirmed using luteinising hormone and follicle stimulating hormone levels in women who had previously undergone hysterectomy, or who presented within 5 years after natural cessation of menstruation.

The oestrogen receptor status (ER) of primary tumour tissue had been documented in 109 patients; 51% were ER positive (greater than 5 $\text{fmol mg}^{-1}$ cytosol protein). ER estimations were performed at the Tenovus Institute, Cardiff, using the dextran coated charcoal method (Nicholson *et al.*, 1981).

All patients were staged before treatment in an advanced breast cancer clinic. All but six patients had received no previous endocrine treatment for overt metastases. Additional symptomatic treatments were prescribed throughout the study which included systemic analgesics, steroids and/or bronchodilators for lung metastases, and radiotherapy for skin ulceration or localised bone pain. Patients unassessable to hormonal therapy by either the type of presenting disease or the addition of local treatments were not included in the study. Sixteen patients were excluded from analysis as sequential serum samples were not available because of early death. The patients studied, therefore, represented the great majority of those attending one unit over a 2-year period, who received endocrine therapy for assessable metastatic disease.

Initial staging included a full clinical examination, documentation of the Karnofsky performance status (Karnofsky & Burchenal, 1948) and accurate measurement of local and regional disease, with photography when considered helpful for later evaluation of response. Radiological assessment consisted of lateral and antero-posterior views of the skull, chest, dorso-lumbar spine, pelvis and upper femora (limited skeletal survey). Painful sites of disease at other locations were X-rayed when clinically indicated. Isotope bone, liver and brain scans were requested only if clinically indicated.

Haematological assessment consisted of measurement of a full blood count, urea and electrolytes, serum calcium and albumin, the erythrocyte sedimentation rate and liver function tests (gamma-glutamyl transaminase, alanine transferase, alkaline phosphatase and bilirubin). In addition, several other 'tumour related markers' were assessed sequentially throughout active treatment (Table I). These included two oncofetal proteins, serum carcinoembryonic antigen (CEA) and ferritin; two acute phase proteins, c-reactive protein (CRP) and orosomucoid; and the hormone beta human chorionic gonadotrophin (B-HCG). Calcium excretion (CAE) and the hydroxyproline creatinine ratio (OHP/CR) were

*Present address: North Staffordshire Royal Infirmary, Princes Road, Stoke on Trent, UK.
Correspondence: R.W. Blamey
Received 4 October 1988; and in revised form 6 July 1989.

**Table I** Tumour markers studied

| | *Upper limit of normal adopted for study* |
|---|---|
| *Serum (all patients)* | |
| Carcinoembryonic antigen[a] | 6 ng ml$^{-1}$ |
| Ferritin[a] | 220 µg l$^{-1}$ |
| C-Reactive protein[a] | 10 mg l$^{-1}$ |
| Orosomucoid[a] | 1.2 g l$^{-1}$ |
| Erythrocyte sedimentation[a] | 20 mm h$^{-1}$ |
| Gamma-glutamyl transpeptidase | 50 U l$^{-1}$ |
| Alanine aminotransferase | 50 U l$^{-1}$ |
| Alkaline phosphatase | 300 U l$^{-1}$ |
| Beta human chorionic gonadotrophin | |
| *Urine (bone metastases only)* | |
| Hydroxyprolene/creatinine ratio | |
| Calcium excretion | |

[a]Tumour markers combined to form therapeutic index for response.

recorded throughout treatment in the urine of a subgroup of patients presenting with bone metastases ($n = 152$ for CAE, $n = 94$ for OHP/CR).

### *Controls*

Marker levels were established in women with benign breast disease attending a diagnostic breast clinic (controls, $n = 55$ for analysis of CEA and ferritin; $n = 25$ for analysis of acute phase proteins). These women had a mean age of 49 years (range 28–85) and presented with histologically confirmed benign breast lumps, breast pain or cysts.

The pre-treatment concentration of each marker in stage IV disease was also compared with that found in 87 patients with untreated stage III breast cancer (see Results).

### *Follow-up*

Patients were reviewed at 1–3 monthly intervals. Those with bone metastases provided a fasting, early morning urine sample for the measurement of CAE and the OHP/CR ratio. The Karnofsky performance status was recorded at each visit, venous blood was withdrawn for measurement of routine haematological parameters and additional serum was centrifuged, aliquoted and stored at −170°C for later evaluation of the remaining markers.

Limited skeletal radiology was repeated after 3–4 months and after 6 months of treatment. At the first clinical sign of objective progression patients were prescribed alternative treatments and therefore removed from study. All assessments of clinical progress were performed without referral to biochemical data, and both the initial documentation of disease status and subsequent assessments of clinical progress were performed by the same observer at each visit (M.R.W.).

### *Assessment of clinical response*

UICC criteria have been strictly applied with the British Breast Group stipulation that any remission should be of at least 6 months' duration to classify as response (British Breast Group, 1974; Hayward *et al.*, 1977). These criteria require a 50% reduction in measurable tumour or objective signs of response in evaluable, but non-measurable, sites of disease (e.g. lung or bone metastases).

Clinical response has been categorised as 'disease progression' (greater than 25% increase in the bidimensional product of measureable tumour or the development of new lesions), 'objective response' (greater than 50% reduction in the size of measurable tumour with no new lesions) or 'disease stabilisation' (no new lesions and any alteration in tumour size lying between these two extremes). In this study response of less than 6 months' duration has been classified as disease progression. External review of response was performed by Dr A. Howell, Christie Hospital, Manchester.

### *Assay techniques*

Routine biochemical and haematological parameters were measured using standard techniques.

CEA was measured using a monoclonal radiometric assay (Tandem-R CEA, kindly provided by Hybritech UK Ltd). The intra- and inter-assay coefficients of variation were 4.6–7.6% and 6.9–7.2%, respectively.

Serum concentrations of ferritin were measured using a solid phase, two site radioimmunoassay. Standards were prepared in ferritin free serum and calibrated against a WHO ferritin reference preparation. Standards covered the range of 0–1000 µg l$^{-1}$. The estimated intra- and inter-assay coefficients of variation, over the working range, were 3.7–5.9% and 4.6–6.0%, respectively.

Concentrations of the beta subunit of human chorionic gonadotrophin were measured by radioimmunoassay using an antiserum raised in rabbit against B-HCG. Standards were calibrated against the First International Reference Preparation (kindly provided by the National Institute of Biological Standards and Control, London) and were prepared to cover the range 0–640 IU l$^{-1}$. The intra- and inter-assay coefficients of variation were 4.7–6.1% and 5.4–8.8%, respectively.

Serum concentrations of CRP were measured using a turbidometric technique on a centrifugal fast analyser (Centrifichem Roche). Changes in optical density were monitored, using an on-line computer which calculated CRP concentrations (O'Callaghan *et al.*, 1984).

Orosomucoid was measured by immuno-turbimetry using a centrifugal fast analyser (Centrifichem 400). Standards were prepared from a commercially available serum (Behring Standard Human Serum, ORDT 06/07). Between batch precision for the assay was 7% at a concentration of 0.65 g l$^{-1}$.

Urinary OHP was estimated in duplicate using Hypronosticon Kits (Organon).

### *Analysis of results*

Pre-treatment concentrations of each tumour marker were compared in individual patients with values recorded after 1–2, 3–4 and 5–7 months of treatment.

For each marker the correlation between alterations in concentration and clinical assessments of response was examined only in patients who presented with, or developed concentrations exceeding predefined levels; the proportion of patients that this represented for each marker is shown in the results. The object of this was to examine markers in each individual only when concentrations were well above those found in the majority of women without advanced disease; concentrations above this level may then be assumed to be due to the tumour. Such concentrations were seen in only 2–8% of women in the control groups. The 'cut-off' chosen for analysis also depended upon the shape of the distribution plots for each marker.

Patients maintaining concentrations within the 'normal' range have been considered unassessable for the marker in question. Above this level concentrations were classified as increasing or decreasing when they altered by more than 10% during treatment.

For statistical analysis patients with rising marker concentrations were combined with those in whom concentrations remained stable. Patients with no change in clinical disease status over 6 months, were combined with those showing objective response. Statistical significance was assessed using the $\chi^2$ test with Yates' correction.

Finally, results of five markers were combined to form a 'therapeutic index', in order to improve the discrimination of response after 3–4 months of treatment.

### Results

### *Patient details and therapeutic response*

One hundred and six patients presented with bone or lung metastases alone ($n = 69$ and $n = 37$, respectively). Thirtyeight presented with both bone and lung metastases, and 35 presented with other sites of visceral involvement (mainly hepatic). Twenty-three per cent of patients (41/179) were found to be premenopausal at the presentation of advanced disease.

The overall response rate was 26% ($n = 47$) using UICC criteria after 6 months. In 17% ($n = 31$) disease remained static for 6 months and in 56% ($n = 101$) disease progressed. Disease either responded or remained static in 64% (36/56) of ER positive patients and in 26% (14/53) of ER negative patients.

*Correlation between therapeutic response and alterations in individual marker concentrations*

In only five markers did appropriate alterations in concentration correlate with UICC response to a degree that could be usefully employed in clinical practice (Table II). For the remaining markers the correlation was weak and these latter results are not discussed further in this report.

In total, 579 serum samples were obtained for the analysis of tumour markers in patients with stage IV disease. These included 179 samples obtained immediately before treatment, 132 obtained after 1 or 2 months (from 128 patients), 131 after 3 or 4 months (from 128 patients) and 137 obtained after 5–7 months of treatment (from 114 patients).

Only the first recorded assay result has been considered for analysis when these were duplicated in individual patients during a single interval of treatment. Individual results for the five markers, recorded simultaneously on the same serum sample, were unavailable on several occasions; four pre-treatment results and 10 follow-up results were not available for ESR, three follow-up results for CRP, and one for both orosomucoid and ferritin.

For each marker, the pre-treatment concentration in stage IV disease was compared with that found both in disease-free women attending a diagnostic breast clinic and in a series of patients presenting with stage III disease, in order to establish upper limits of normal.

*Carcinoembryonic antigen* Only one control patient presented with a CEA concentration in excess of 6 ng $ml^{-1}$, compared with 18% (16/87) of those with stage III disease and 49% (88/179) of those presenting with stage IV disease (Figure 1, median concentrations illustrated). Alterations in CEA during treatment have been examined only when concentrations exceeded 6 ng $ml^{-1}$.

**Figure 1** Carcinoembryonic antigen in controls and at presentation of stage III and IV disease: median concentrations illustrated.

One hundred and twenty-eight stage IV patients had CEA concentrations measured before and after 1 or 2 months of treatment. Sixty-three (49% of the total) were assessable employing a CEA concentration in excess of 6 ng $ml^{-1}$. One hundred and twenty-eight patients had CEA measurements repeated after 3 or 4 months of treatment, 71 (55%) were biochemically assessable as above. After 5–7 months of treatment, 65 of 114 patients (57%) wre biochemically assessable. A highly significant association was found between the clinical assessment of response after 6 months, and changes in tumour marker concentration (above 6 ng $ml^{-1}$) during all three periods of treatment (Table II: pre-treatment *vs* 1–2 months, $\chi^2 = 15$, 1 d.f.; *vs* 3–4 months, $\chi^2 = 33.8$, 1 d.f.; *vs* 5–7 months, $\chi^2 = 28.16$, 1 d.f.).

*Ferritin* Only three control patients presented with serum concentrations in excess of 220 $\mu g\,l^{-1}$, compared with 7% (6/87) of stage III patients and 30% (53/179) of those with stage IV disease (Figure 2). Alterations in serum ferritin in

**Table II** Alterations in individual serum marker concentrations during treatment versus therapeutic response

| Tumour marker | UICC response at 6 months | *Marker concentrations pre-treatment versus 1–2 months treatment* | | | *Marker concentrations pre-treatment versus 3–4 months treatment* | | | *Marker concentrations pre-treatment versus 5–7 months treatment* | | |
|---|---|---|---|---|---|---|---|---|---|---|
| | | *Decrease* | *Stable* | *Increase* | *Decrease* | *Stable* | *Increase* | *Decrease* | *Stable* | *Increase* |
| CEA | Response | 15 | 2 | 3 | 22 | 1 | 1 | 22 | 0 | 3 |
| | Static | 5 | 1 | 3 | 8 | 1 | 2 | 11 | 2 | 2 |
| | Progression | 6 | 9 | 19 | 5 | 2 | 29 | 3 | 1 | 21 |
| | | | ($\chi^2 = 15.1$; 1.d.f.)* | | | ($\chi^2 = 33.8$; 1.d.f.)* | | | ($\chi^2 = 28.16$; 1.d.f.)* | |
| Ferr. | Response | 11 | 4 | 1 | 13 | 2 | 0 | 18 | 1 | 2 |
| | Static | 5 | 0 | 1 | 5 | 1 | 1 | 8 | 0 | 0 |
| | Progression | 5 | 4 | 19 | 6 | 0 | 21 | 1 | 3 | 15 |
| | | | ($\chi^2 = 15.1$; 1.d.f.)* | | | ($\chi^2 = 23.4$; 1.d.f.)* | | | ($\chi^2 = 23.0$; 1.d.f.)* | |
| CRP | Response | 21 | 0 | 1 | 22 | 0 | 0 | 24 | 0 | 1 |
| | Static | 9 | 0 | 2 | 13 | 0 | 3 | 14 | 1 | 3 |
| | Progression | 10 | 3 | 24 | 9 | 2 | 24 | 6 | 0 | 18 |
| | | | ($\chi^2 = 26.52$; 1.d.f.)* | | | ($\chi^2 = 30.82$; 1.d.f.)* | | | ($\chi^2 = 24.7$; 1.d.f.)* | |
| Oroso. | Response | 26 | 2 | 2 | 25 | 1 | 2 | 29 | 3 | 1 |
| | Static | 11 | 1 | 0 | 13 | 1 | 3 | 15 | 1 | 2 |
| | Progression | 16 | 13 | 17 | 22 | 5 | 26 | 11 | 7 | 15 |
| | | | ($\chi^2 = 23.87$; 1.d.f.)* | | | ($\chi^2 = 26.4$; 1.d.f.)* | | | ($\chi^2 = 22.6$; 1.d.f.)* | |
| ESR | Response | 23 | 2 | 5 | 27 | 0 | 6 | 31 | 1 | 4 |
| | Static | 11 | 3 | 1 | 15 | 0 | 1 | 15 | 2 | 3 |
| | Progression | 17 | 6 | 23 | 11 | 5 | 32 | 14 | 0 | 21 |
| | | | ($\chi^2 = 12.24$; 1.d.f.)* | | | ($\chi^2 = 36.09$; 1.d.f.)* | | | ($\chi^2 = 15.21$; 1.d.f.)* | |

Statistical significance assessed by combining 'response' with 'static', and 'stable' with 'increase' *$P<0.001$.

**Figure 2** Ferritin in controls and at presentation of stage III and IV disease: median concentrations illustrated.

stage IV disease have been examined only above $220\,\mu g\,l^{-1}$.

One hundred and twenty-eight patients had ferritin levels recorded before and after 1 or 2 months of treatment. Fifty (39% of the total) were assessable using concentrations above $220\,\mu g\,ml^{-1}$. In one hundred and twenty-seven patients ferritin was measured before and after 3 or 4 months of treatment; 49 (39%) were biochemically assessable as above. In 114 patients ferritin was measured before and after 5–7 months of treatment, 48 (42%) were biochemically assessable.

Again, during each interval of treatment, a highly significant association existed between alterations in serum ferritin (above $220\,\mu g\,ml^{-1}$) and clinical assessments of response after 6 months (Table II: pre-treatment *vs* 1–2 months, $\chi^2 = 15.1$, 1 d.f.; *vs* 3–4 months, $\chi^2 = 23.4$, 1 d.f.; *vs* 5–7 months, $\chi^2 = 23$, 1 d.f.).

*C-reactive protein* Only one tumour-free patient presented with a CRP concentration in excess of $10\,mg\,l^{-1}$, compared with 13% (11/87) of those with stage III disease and 53% (94/179) of those presenting with stage IV disease (Figure 3). Thus, alterations in CRP have been examined only when concentrations exceeded $10\,mg\,l^{-1}$.

One hundred and twenty-seven patients with distant metastases had CRP concentrations measured before and after one or two months of treatment, of whom 70 patients (55% of the total) were assessable employing CRP concentrations in excess of $10\,mg\,l^{-1}$. One hundred and twenty-six patients had CRP concentrations measured before and after 3 or 4 months of treatment, 73 (58%) were assessable as above. In 114 patients CRP was measured before and after 5–7 months of treatment, 67 (59%) were biochemically assessable.

A highly significant association existed between therapeutic response (UICC) and alterations in CRP concentration during the three time intervals of treatment (Table II: pre-treatment *vs* 1–2 months, $\chi^2 = 26.52$, 1 d.f.; *vs* 3–4 months, $\chi^2 = 30.82$, 1 d.f.; *vs* 5–7 months, $\chi^2 = 24.7$, 1 d.f).

*Orosomucoid* Only two control patients presented with an orosomucoid concentration in excess of $1.2\,g\,l^{-1}$, compared with 17% (15/87) of those with stage III disease and 61% (110 179) of those with stage IV disease (Figure 4). Altera-

**Figure 3** C-reactive protein in controls and at presentation of stage III and IV disease: median concentrations illustrated.

tions in concentration during treatment have been examined only when levels exceeded $1.2\,g\,l^{-1}$.

One hundred and twenty-seven patients with distant metastases had CRP concentrations measured before and after one or two months of treatment, of whom 70 patients (55% of the total) were assessable employing CRP concentrations in excess of $10\,mg\,l^{-1}$. One hundred and twenty-six patients had CRP concentrations measured before and after 3 or 4

**Figure 4** Orosomucoid in controls and at presentation of stage III and IV disease: median concentrations illustrated.

months of treatment, 73 (58%) were assessable as above. In 114 patients CRP was measured before and after 5–7 months of treatment, 67 (59%) were biochemically assessable.

A highly significant association existed between alterations in marker concentrations during each interval of treatment, and therapeutic response assessed using UICC criteria after six months (Table II: pre-treatment *vs* 1–2 months, $\chi^2 = 23.87$, 1 d.f.; *vs* 3–4 months, $\chi^2 = 26.4$, 1 d.f.; *vs* 5–7 months, $\chi^2 = 22.6$, 1 d.f).

*Erythrocyte sedimentation rate* The ESR was greater than 20 mm $h^{-1}$ in only two of 50 tumour-free patients attending a diagnostic breast clinic (not illustrated). In contrast, the ESR was greater than 20 mm $h^{-1}$ in 33% (26/79) of patients presenting with stage III disease and in 66% (115/175) of those presenting with stage IV disease (Figure 5). Alterations in the ESR have been examined only above 20 mm $h^{-1}$.

One hundred and twenty-one stage IV patients had ESR measurements before and after 1 or 2 months of treatment. Ninety-one (75% of the total) were assessable employing an ESR of greater than 20 mm $h^{-1}$. One hundred and twelve patients had the ESR measured before and after 3 or 4 months of treatment, 97 (87%) were assessable. In 111 patients the ESR was measured before and after 5–7 months of treatment, 91 (82%) were assessable employing an ESR greater than 20 mm $h^{-1}$.

Again, a highly significant association existed between alterations in the ESR during each interval of treatment and therapeutic response assessed after 6 months using UICC criteria (Table II: pre-treatment *vs* 1–2 months, $\chi^2 = 12.24$, 1 d.f.; *vs* 3–4 months, $\chi^2 = 36.09$, 1 d.f.; *vs* 5–7 months, $\chi^2 = 15.21$, 1 d.f).

**Figure 5** ESR at presentation of stage III and IV disease: median concentrations illustrated.

*Combining markers to assess therapeutic response after 3–4 months of treatment*

From the results of individuals tumour markers it can be seen that the accuracy with which each marker was able to assess response, was highest during the 3–4 month interval of treatment.

In 127 patients the results of all five markers were available during this interval of treatment. Patients were considered biochemically unassessable when the concentrations of all five markers remained below the minimum levels adopted for analysis. This occurred in 10 patients (8% of the total). Of the remaining 117 biochemically assessable patients, 37 responded to treatment (UICC criteria), in 20 disease remained static for 6 months and in 60 disease progressed.

A 'therapeutic index' has been devised by allocating points according to a rise or a fall in each marker concentration above defined levels, as shown in Table III.

Several factors were taken into consideration when constructing the index and different weighting was applied to appropriate alterations in each marker. Progressing disease was associated with high but stable marker concentrations (score + 1). However, high, stable marker concentrations did not detect disease progression as accurately as increasing marker concentrations (score + 2). The predictive value for disease progression of increasing concentrations of ferritin, the ESR and acute phase proteins (score + 2) was higher than the predictive value for non-progressing disease of falling concentrations (score − 1). Finally, appropriate alterations in CEA were equally effective to confirm progressing and non-progressing disease (score + 2 and − 2, respectively).

When all five markers increased in concentration by more than 10%, a maximum index score of + 10 was achieved. Conversely, when all five markers were abnormal at presentation and decreased by more than 10%, a minimum index score of − 6 was achieved.

The distribution of index scores at 3–4 months is shown after subgrouping patients according to UICC assessments of response at 6 months (Figure 6). By employing a 'cut-off' lying

**Figure 6** Biochemical index scores after 3–4 months.

**Table III** Allocation of scores towards a biochemical index for response (values are index scores)

| | *Tumour marker concentrations during treatment* | | | |
|---|---|---|---|---|
| | *Remain within normal limits* | *Decrease by 10%* | *Remain stable* | *Increase by 10%* |
| CEA (>6 ng $ml^{-1}$) | 0 | − 2 | + 1 | + 2 |
| Ferritin (>220 μg $l^{-1}$) | 0 | − 1 | + 1 | + 2 |
| Orosomucoid (>1.2 g $l^{-1}$) | 0 | − 1 | + 1 | + 2 |
| CRP (>10 mg $l^{-1}$) | 0 | − 1 | + 1 | + 2 |
| ESR (>20 mm $h^{-1}$) | 0 | − 1 | + 1 | + 2 |

between index scores of 0 and + 1, the sensitivity that a low index score has for objective response (or disease stabilisation for 6 months) is 89.5% (51/57), with a specificity of 78% (47/60) (Table IV, group A; Figure 7).

When patients with 'marked biochemical change' are considered separately, (index scores greater than + 2 or less than − 2), the sensitivity for response/stasis increases to 92% (34/37) with a specificity of 100% (38/38) (Table IV, group B; Figure 8). Seventy-five patients (59% of the total) developed 'marked biochemical' responses to treatment.

### Discussion

Significant advances have been made in the treatment of malignant diseases where specific markers are able to monitor tumour burden. Two examples are the use of B-HCG assays in choriocarcinoma (Begent & Bagshaw, 1982) and measurements of B-HCG and alpha fetoprotein in patients receiving treatment for testicular teratoma (Lange *et al.*, 1976; Schultz *et al.*, 1978; Lange, 1982). These advances are dependent in part upon the ability to assess changes in tumour bulk at an early stage, which allows appropriate alteration of treatment.

This work seeks a marker of tumour burden for advanced breast cancer so that similar priniciples can be applied. A single marker with the desired specificity for response has not been found, even when each marker was examined at high serum concentrations. However, we have found that a combination of five markers (at high serum concentration) is able accurately to reflect therapeutic response of 6 months' duration. The five assays are easily performed, and their combination is able to discriminate between response groups at a relatively early stage of treatment.

Each marker has been considered only at high serum concentrations, to limit interference from factors independent of tumour burden. Many previous studies have examined tumour markers in advanced breast cancer with the aim of assessing response objectively during early treatment. The majority of these reports can be criticised as the patients studied were few in number, only single markers were examined, marker concentrations remained within normal limits in many patients analysed, and clinical response criteria were often inadequate.

Several problems are encountered when employing clinical criteria to assess response retrospectively. They require extensive radiology and repeated measurements of tumour deposits, making any assessment both difficult and time consuming. As a result, clinical signs of response may be misinterpreted, especially when assessments are made on plain X-rays.

Bone is the commonest site for distant metastases from breast cancer and it is particularly in this situation that controversy exists over the current criteria for response (Lipshitz & Hortobagyi, 1981; Coombes *et al.*, 1983; Hortobagyi *et al.*, 1984). The development of sclerosis within lytic metastases is regarded as a prerequisite for therapeutic response. This is often slow to develop and may be absent despite unequivocal evidence of response in other sites. Furthermore, the emergence of blastic metastases may indicate either response (DeMartini *et al.*, 1983) or progression of disease. As radiological evidence of response may take many months to appear, it is frequently of limited value to guide treatment in practice.

In this study, patients with static disease (for a minimum duration of 6 months) were combined with those showing objective clinical evidence of response. Previous studies have indicated that these patients fair as well as responders with respect to survival (Howell *et al.*, 1984; Williams *et al.*, 1986). Biochemical responses to treatment, assessed using this combination of markers, were similar in patients showing partial response or static disease. In contrast, patients with progressive disease showed markedly different biochemical profiles. It is submitted that the employment of clinical criteria alone is inadequate to detect response in many patients found to have stable disease on clinical grounds.

This biochemical index of response was devised retrospectively. It will require prospective testing on a further series of

**Table IV** Biochemical index scores after 3–4 months' treatment

| | *Group A* | | *Group B* | |
|---|---|---|---|---|
| *UICC assessment* | *≤0* | *>0* | *<−2* | *>+2* |
| Response | 35 | 2 | 23 | 0 |
| Static | 16 | 4 | 11 | 3 |
| Progression | 13 | 47 | 0 | 38 |
| | ($\chi^2$ = 51.54; 1 d.f.)* | | ($\chi^2$ = 70.05; 1 d.f.)* | |
| | (*P*<0.0001) | | (*P*<0.0001) | |

*'Responding' and 'static' patients combined for analysis. Group A: all assessable patients (*n* = 117, 91% of total); group B: marked biochemical changes (*n* = 75, 59% of total).

**Figure 7** Distribution of index scores in all patients after 3–4 months.

**Figure 8** Distribution of index scores in patients with 'marked biochemical change' after 3–4 months.

patients to assess its reproducibility. Where biochemical and clinical assessments of response are at variance, both criteria should be evaluated against other parameters such as survival. These studies are currently in progress and if the index is confirmed as correlating strongly with an assessment of response based on UICC criteria, then the biochemical assessment has several important advantages. These include its simplicity, objectivity and ability to assess response at an early stage and thus guide treatment. There are also economic advantages, as marker estimations amount to only a small proportion of the costs of limited skeletal surveys.

The UICC criteria were designed to be employed retrospectively and in the context of therapeutic trials only. They are often not adequate for ongoing assessments of therapeutic response during treatment. Provided this biochemical measurement of response proves reproducible, it might be possible to replace clinical criteria for response by this simple and truly objective alternative.

During the period of study M.R.W. was supported by a generous grant provided by the Tenovus Institute, Cardiff. The authors would like to thank the following for their co-operation in the analysis of tumour markers: Miss Norah Richards, Dr R. Powell, Mr K. Quilter and Dr G. Walker, Departments of Immunology and Clinical Chemistry, Queens Medical Centre, Nottingham, and Miss Julie Haynes, Department of Surgery, Nottingham.

### References

BEGENT, R.M.J. & BAGSHAW, K.D. (1982). The management of high risk choriocarcinoma. *Semin. Oncol.*, **9**, 198.

BORTHWICK, N.M., WILSON, D.W. & BELL, P.A. (1977). Carcinoembryonic antigen (CEA) in patients with breast cancer. *Eur. J. Cancer*, **33**, 171.

BRITISH BREAST GROUP (1974). Assessment of response to treatment in advanced breast cancer. *Lancet*, **ii**, 38.

CAFFIER, H. & BRANDAU, H. (1983). Serum tumor markers in metastatic breast cancer and course of disease. *Cancer Detection Prevention*, **6**, 451.

CAMPBELL, F.C., BLAMEY, R.W., WOOLFSON, A.M.J., ELSTON, C.W. & HOSKIN, D.J. (1983). Calcium excretion (CAE) in metastatic breast cancer. *Br. J. Surg.*, **70**, 202.

CHU, T.M. & NEMOTO, T. (1973). Evaluation of carcinoembryonic antigen in human mammary carcinoma. *J. Natl Cancer Inst.*, **51**, 1119.

COOMBES, R.C., POWLES, T.J., GAZET, J.C. & 10 others (1977). A biochemical approach to the staging of human breast cancer. *Cancer*, **40**, 937.

COOMBES, R.C., DADY, P., PARSONS, C. & 4 others (1983). Assessment of response of bone metastases to systemic treatment in patients with breast cancer. *Cancer*, **52**, 610.

COVE, D.H., WOODS, K.L., SMITH, S.C.H. & 4 others (1979). Tumour markers in breast cancer. *Br. J. Cancer*, **40**, 710.

COWEN, D.M., SEARLE, F., WOOD, A.M. & 4 others (1978). Multivariate biochemical indicators of breast cancer: an evaluation of their potential in routine practice. *Eur. J. Cancer*, **14**, 888.

DEMARTINI, A.L., BUZDAR, A.U. & BLUMENSCHEIN, G.R. (1983). Osteoblastic metastatic disease as a therapeutic response to adjuvant chemotherapy in breast cancer. *J. Surg. Oncol.*, **23**, 32.

HAAGENSON, D.E., KISTER, S.J., VANDERVOORDE, J.P. & 4 others (1978). Evaluation of carcinoembryonic antigen as a plasma monitor for human breast cancer. *Cancer*, **42**, 1512.

HAAGENSON, D.E., BARRY, W.F., MCCOOK, T.A., GIANNOLA, J., AMMIRATA, S. & WELLS, S.A. (1980). The value of serial plasma levels of carcinoembryonic antigen and gross cyst disease fluid protein in patients with breast carcinoma and osseus metastases. *Ann. Surg.*, **191**, 599.

HAYWARD, J.L., CARBONE, P.P., HEUSON, J.C., KUMAOKA, S., SEGALOFF, A. & RUBENS, R.D. (1977). Assessment of response to therapy in advanced breast cancer. *Cancer*, **39**, 1289.

HEIM, M.E., SAUER, B., SCHNITZLER, G., HAUX, P. & FELDMANN, U. (1984). Bedeutung des carcinoembryonalen antigens fur die therapiekontrolle von fortgeschrittenen mammakarzinomen. *Tumor Diag. Ther.*, **5**, 220.

HORTOBAGYI, G.N., LIBSHITZ, H.I. & SEABOLD, J.E. (1984). Osseus metastases of breast cancer. Clinical, biochemical, radiographic and scintigraphic evaluation of response to therapy. *Cancer*, **53**, 577.

HOWELL, A., BARNES, D.M., HARLAND, R.N.L. & 6 others (1984). Steroid-hormone receptors and survival after first relapse in breast cancer. *Lancet*, **i**, 588.

KARNOFSKY, D.A. & BURCHENAL, J.H. (1948). In *Evaluation of Chemotherapeutic Agents*, Ed. MacLeod, C.M. (ed.).

KRIEGER, G., WANDER, H.E., PRANGEN, M., BANDLOW, G., BEYER, J.H. & NAGEL, G.A. (1983). Bestimmung des carcinoembryonalen antigens (CEA) zur voraussage des therapieerfolges beim metastasierenden mammarkarzinom. *Dtsch. Med. Wschr.*, **108**, 610.

LAMERZ, R., LEONHARDT, A., EHRHART, E. & LIEVEN, H.V. (1980). Serial carcinoembryonic antigen (CEA) determinations in the management of metastatic breast cancer. *Oncodevel. Biol. Med.*, **1**, 123.

LANGE, P.H., MCINTYRE, K.R., WALDMAN, T.A., HAKALA, T.R. & FRALEY, E.E. (1976). Serum alphafetoprotein and human chorionic gonadotrophin in the diagnosis and management of nonseminomatous germ-cell testicular cancer. *N. Engl. J. Med.*, **295**, 1237.

LANGE, P. (1982). Testicular cancer markers. In *Human Cancer Markers*, Sell, S. & Wahren, B. (eds) p. 265. Humana Press:

LEE, Y.-T.N. (1983). Serial tests of carcinoembryonic antigen in patients with breast cancer. *Am. J. Clin. Oncol.*, **6**, 287.

LIBSHITZ, H.I. & HORTOBAGYI, G.N. (1981). Radiographic evaluation of therapeutic response in bony metastases of breast cancer. *Skeletal Radiol.*, **7**, 159.

NICHOLSON, R.I., CAMPBELL, F.C., BLAMEY, R.W., ELSTON, C.W., GEORGE, D. & GRIFFITHS, K. (1981). Steroid receptors in early breast cancer: value in prognosis. *J. Steroid Biochem.*, **15**, 193.

O'CALLAGHAN, C., FRANKLIN, P., ELLIOTT, T.S.J., DEVERILL, I., RICHARDS, N. & POWELL, R.J. (1984). C-reactive protein concentrations in neonates: determination by a latex enhanced immunoassay. *J. Clin. Pathol.*, **37**, 1027.

SCHULTZ, H., SELLS, A., NORGAARD-PEDERSON, B. & ARENDS, J. (1978). Serum alphafetoprotein and human chorionic gonadotrophin as markers for the effect of postoperative radiation therapy and/or chemotherapy in testicular cancer. *Cancer*, **42**, 2182.

STEWARD, A.M., NIXON, D., ZAMCHECK, N. & AINSENBERG, A. (1974). Carcinoembryonic antigen in breast cancer patients: serum levels and disease progress. *Cancer*, **33**, 1246.

TORMEY, D.C., WAALKES, T.P., SNYDER, J.J. & SIMON, R.M. (1977*a*). Biological markers in breast cancer. III. Clinical correlations with carcinoembyonic antigen. *Cancer*, **39**, 2397.

TORMEY, D.C., WAALKES, T.P. & SIMON, R.M. (1977*b*). Biological markers in breast cancer. II. Clinical correlation with human chorionic gonadotrophin. *Cancer*, **39**, 2391.

WILLIAMS, M.R., TODD, J.H., NICHOLSON, R.I., ELSTON, C.W. & BLAMEY, R.W. (1986). Survival patterns in endocrine treated advanced breast cancer. *Br. J. Surg.*, **73**, 752.

WOO, K.B., WAALKES, P.T., AHMANN, D.L., TORMEY, D.C., GEHRKE, C.W. & OLIVERIO, V.T. (1978). A quantitative approach to determining disease response during therapy using multiple biological markers. Application to carcinoma of the breast. *Cancer*, **41**, 1685.